# Cutaneous Tuberculosis of Gluteal Region Presenting as a Sinus and Large Cyst: An Unusual Entity

**DOI:** 10.4103/0974-2077.79201

**Published:** 2011

**Authors:** Anupama Gupta, Sunita Gupta, Rikki Singal, Prem Singh, Amit Mittal, Samita Gupta, Raman Gupta

**Affiliations:** 1 *Department of Anatomy, Adesh Institute of Medical Sciences and Research, Bathinda, Punjab, India*; 2 *Department of Medicine, Maharishi Markandeshwer Institute of Medical Sciences and Research, Mullana, Ambala, Haryana, India*; 3 *Department of Surgery, Maharishi Markandeshwer Institute of Medical Sciences and Research, Mullana, Ambala, Haryana, India. E-mail: singalrikki@yahoo.com*; 4 *Department of Pathology, Maharishi Markandeshwer Institute of Medical Sciences and Research, Mullana, Ambala, Haryana, India*; 5 *Department of Radiodiagnosis and Imaging, Maharishi Markandeshwer Institute of Medical Sciences and Research, Mullana, Ambala, Haryana, India*; 6 *Department of Surgery, Adesh Institute of Medical Sciences and Research, Bathinda, Punjab, India*

Sir,

Extra-pulmonary tuberculosis (TB) is a rare form of cutaneous TB and constitutes only about 0.11% to 2.5% of all patients with skin diseases. Infections due to mycobacterium tuberculosis are classified according to the inoculation route.[1,2]

A 35-year-old woman presented with a discharge in the gluteal region since 5 months. She had history of intramuscular injection over the gluteal region from a private practitioner 6 months back, which led to an abscess/ sinus formation. There was yellowish thick discharge, approximately 200-300 ml. Pain in the buttock region was severe in nature in the preceding 2 weeks. History of on-and-off fever was present since 2 months without any cough or vomiting. There was no positive family history of TB. Erythrocyte sedimentation rate was raised (45 mm/h). Mantoux tests and X-rays of chest, spine and pelvis were normal.

On local examination, a small opening was seen over the lateral aspect of the right buttock, from which there was a yellowish discharge. It was sent for bacterial culture, which was found to be negative for tuberculosis. Swelling was felt in the right buttock region of size 10 × 8 cm, which was firm, non-tender and non-mobile in nature. Skin colour and movements of the lower limbs were normal.

**Figure 1:**
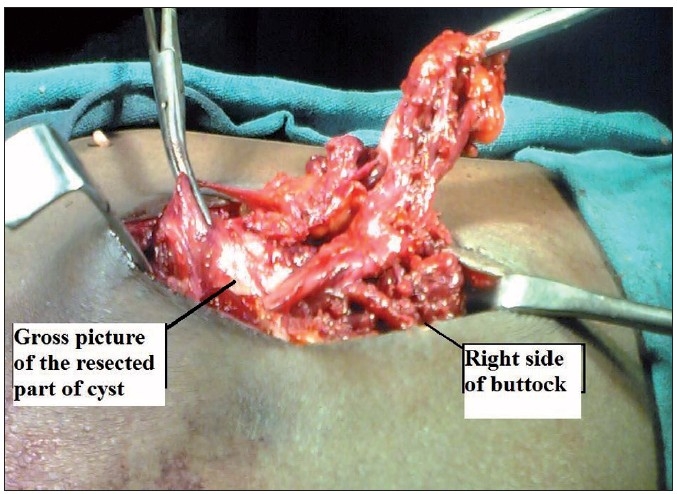
Operative picture of the right gluteal region showing a large cyst

**Figure 2:**
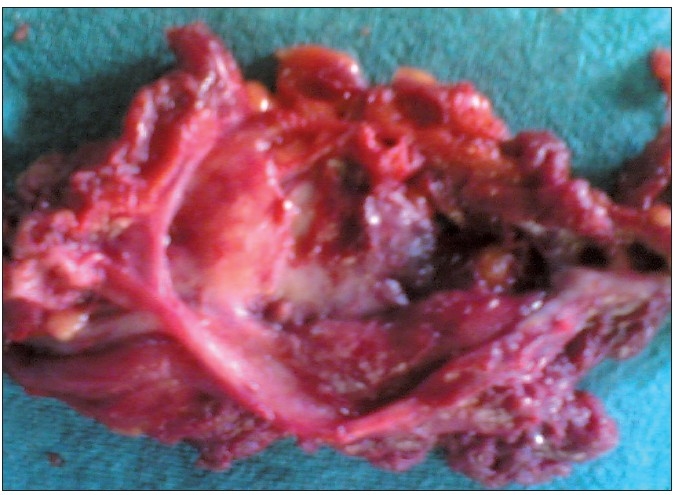
Gross specimen of the cyst showing thickened wall

An elliptical incision was made and we found that the track was going deep into the muscles and a large cyst was present there [Figure 1]. Cyst was of about 10 × 8 cm in size, thick walled, adherent to the gluteal muscles and contained yellowish thick fluid [Figure 2]. Sinus and the entire cyst were excised. Skin was closed primarily, along with negative pressure suction drain. Stitches were removed on the 10^th^day. At the 6-month follow-up, the patient was asymptomatic without any recurrence.

**Figure 3:**
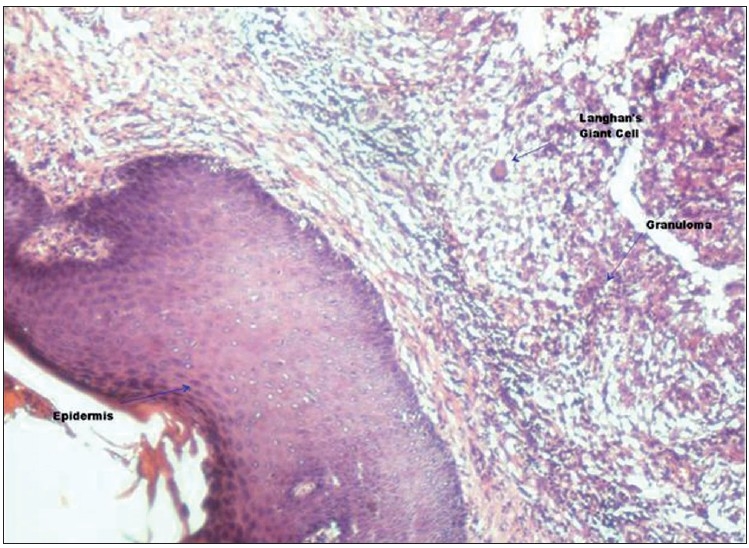
Photomicrograph showing epidermis on the left side and granulomatous infiltrate of Langhan’s giant cells, epithelioid cells, lymphocytes, plasma cells on the right side (H and E, ×100)

**Figure 4:**
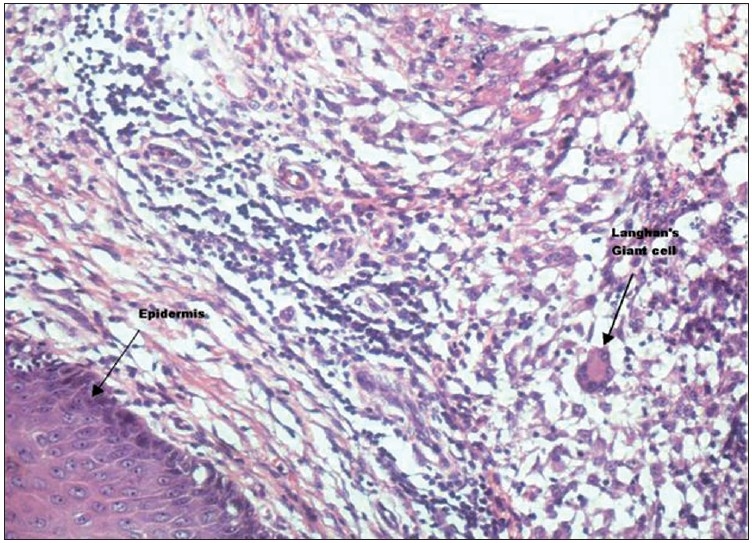
Photomicrograph showing epidermis on the left side and granulomatous infiltrate of Langhan’s giant cells, epithelioid cells, lymphocytes, plasma cells on the right side (H and E, ×200)

**Figure 5:**
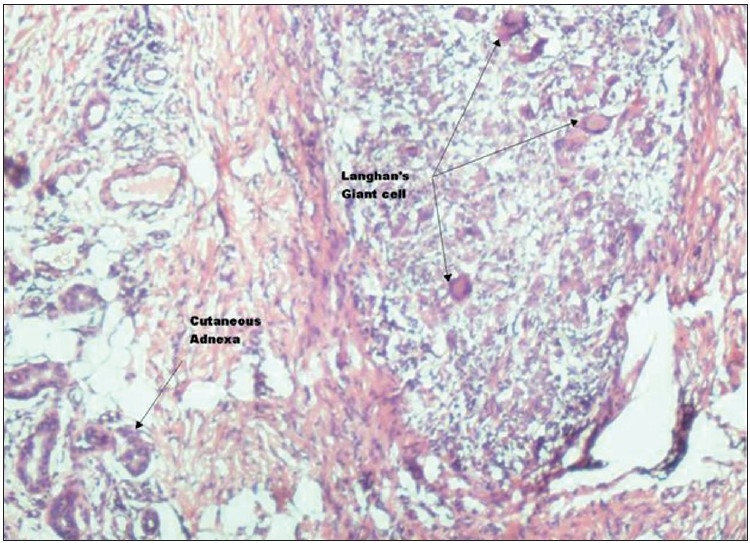
Photomicrograph showing adnexal structures on the left side and granulomatous infiltrate of Langhan’s giant cells, epithelioid cells, lymphocytes, plasma cells on the right side (H and E, 100×)

On microscopic examination, large cyst cavity was seen lined by fibrocollagenous tissue showing infiltration by mononuclear cells and occasional Langhan’s type of giant cells [Figure 3]. Muscular tissue was normal. Sub-epidermal tissue showed dense granulomatous infiltrate consisting of epithelioid cells, plasma cells, lymphocytes and numerous Langhan’s type of giant cells [Figures 4 and 5]. The patient was put on antitubercular therapy – rifampicin, isoniazid, pyrazinamide and ethambutol for the first 6 months; and 2 months later, continued with rifampicin and isoniazid.

A subcutaneous TB associated with cold abscess results from direct extension of an underlying focus such as lymph node, bone or joint to the overlying skin, which presents as firm painless subcutaneous nodules that lead to the formation of ulcers and sinus tracts, as in our case. The areas of predilection are the neck, supraclavicular fossa, axilla and groin.[2,3]

Tubercular abscess usually occurs by direct extension from the neighbouring joint or rarely by haematogenous or lymphatic spread from the infection in pulmonary or extra-pulmonary site, though a primary focus may not be detected in every case.[4] Post-injection tubercular abscesses are very rare and theoretically occur in two ways. Firstly, through a primary inoculation, if the organisms are introduced by contaminated injection material or instrument, which is usually rare. The second and common pathogenesis is seen in patients who have recently contacted primary infection and during this early stage, a number of bacilli reach the blood stream, either directly from the initial focus or via regional lymph node and thoracic duct.[5]

In conclusion, for any swelling/ sinus or cyst at an injection site presenting without any signs of inflammation, and not responding to antibiotics, a possibility of cutaneous tuberculosis should be kept in mind.
